# Accuracy of mammography and ultrasonography and their BI-RADS in detection of breast malignancy

**DOI:** 10.22088/cjim.12.4.573

**Published:** 2021

**Authors:** Naser Ghaemian, Neda Haji Ghazi Tehrani, Mehrdad Nabahati

**Affiliations:** 1 Department of Radiology, Shahid Beheshti Hospital, Babol University of Medical Sciences, Babol, Iran; 2 Student Research Committee, Babol University of Medical Sciences, Babol, Iran

**Keywords:** Ultrasonography, Mammography, Core needle biopsy, Breast malignancy

## Abstract

**Background:**

We aimed to compare the diagnostic accuracy of mammography and ultrasonography and their breast imaging-reporting and data system (BI-RADS) classification versus breast core needle biopsy (CNB) findings in distinguishing the breast masses.

**Methods:**

This cross-sectional study was conducted during 2016-2018 on female patients who were referred to a radiology center in Babol, northern Iran, for routine screening and/or for CNB. Patients underwent sonography and mammography by a senior radiologist. The breast lesions were also evaluated according to BI-RADS classification. CNB was performed on the breast masses by the same radiologist and pathological procedures were performed by an expert pathologist. Descriptive statistics were used to analyze the data.

**Results:**

In total, 213 breast masses were finally assessed, of which 107 (50.2 %) masses were benign and 106 (49.8 %) masses were malignant. The sensitivity for mammography and ultrasound alone was 72.6% and 68.9%, respectively. This rate for combined mammography and ultrasound was 84.9%. About BI-RADS classification, 28 masses were classified as BI-RADS 3, 99 as BI-RADS 4A, 4 as BI-RADS 4B, 18 as BI-RADS 4C, and 64 as BI-RADS 5. BI-RADS 4A had the highest sensitivity (70.1%) among BI-RADS categories. The highest specificity pertained to BI-RADS 3 and 5 (100%) among BI-RADS categories. Also, the highest accuracy was related to BI-RADS 5 (80.3%).

**Conclusion:**

The results of the present study showed that combined mammography and ultrasound had a higher rate of accuracy than mammography or ultrasound alone. Furthermore, the imaging methods BI-RADS classification had an acceptable positive predictive value.

Breast cancer is the most common malignancy and the second leading cause of cancer death among adult women. Every year, more than 2 million new patients of breast cancer are diagnosed worldwide and this rate is rising (1). Iran has a similar condition and breast cancer ranks first among malignancies in women and has been a major cost of the health care system. The age-standardized rate for this disease is 31 per 100,000 people, according to GLOBOCAN 2018 report (2). Therefore, earlier diagnosis of breast malignancies should be noticed to control their outcome in this country. Mainly, women with suspicious symptoms or palpable masses of the breast on clinical examination undergo imaging assessments, including ultrasound and mammography. These non-invasive techniques are the main imaging methods for the evaluation of breast abnormalities. Mammography is used in both the screening and diagnosis of breast masses. Finally, suspicious imaging findings need pathological assessments for the definite diagnosis (3, 4).

Considering the increasing rate of newly diagnosed cases in breast imaging, a collaboration between radiologists and pathologists seems necessary in the evaluation of the consistency of radiologic and pathological findings, so that the correct and appropriate approach is considered. In this regard, the American College of Radiology developed a standardized format and terminology named breast imaging-reporting and data system (BI-RADS). BI-RADS is the most important part of an imaging report. In this system, all reports should start with a description of the overall composition of the breast. BI-RADS includes seven categorizations from 0 to 6, and the higher number is in favor of malignancy (5, 6). There is no extensive literature regarding the accuracy of mammography and ultrasonography and their BI-RADS classification in differentiating malignant from benign breast masses, especially in our region. Therefore, we aimed to investigate the accuracy of sonography and mammography features and their BI-RADS scales based on pathology findings in the diagnosis of breast malignancy. This study can help better screening and earlier diagnosis of breast masses and prevention of unnecessary surgeries, and also can be efficient in the follow-up of the patients with breast malignancy.

## Methods

**Locations and patients: **This cross-sectional study was conducted during 2016-2018 on female patients who were consecutively referred to a radiology center in Babol, northern Iran, for routine screening and/or for core needle biopsy (CNB). The decision in the indication for CNB was made through consensus between the physicians/surgeons and the radiologist, based on the patients' history (breast pain, nipple discharge, breast mass, breast asymmetry, nipple retraction, lymphadenopathy, skin lesions), physical examination (e.g., lymphadenopathy, breast mass) and/or imaging findings (e.g., focal asymmetry in the breast). Patients whose BI-RADS was 3, 4, or 5 underwent CNB. Biopsy in patients with BI-RADS 3 was done based on their request or the physician's decision. All patients underwent CNB for the first time. Patients whose CNB results were not available were excluded from the study.

**Imaging and core needle biopsy: **Patients underwent sonography (Philips iU22 Ultrasound Machine, Philips Healthcare, Best, Netherlands) and mammography (Mammomat Inspiration, Siemens, Erlangen, Germany) by a senior radiologist with more than 20 years of experience in performing the procedure. Those patients aged ≥40 years old who had previous mammographic reports underwent mammography again. Based on the sonography, a mass was considered suspicious for malignancy if the following features were found (7-9): irregular margin, microlobulated, posterior shadow, taller than wide, irregular shape, hypoechoic, and heterogeneous lesions. Based on mammography, the following features were considered suspicious (7-9): speculated, microlobulated, microcalcification, and hyperdense lesions.

In the following, a combination of sonography and mammography BI-RADS scores was calculated for each patient and the highest BI-RADS classification was taken into account for further investigations. BI-RADS classification was defined as follows (10):

- BI-RADS 3 (probably benign): ≤ 2% malignancy risk
- BI-RADS 4A (low suspicion): >2% to ≤10% malignancy risk
- BI-RADS 4B (moderate suspicion): >10% to ≤50% malignancy risk
- BI-RADS 4C (high suspicion): > 50% to < 95% malignancy risk
- BI-RADS 5 (probably malignant): ≥ 95% malignancy risk

**Biopsy**: CNB was performed on the breast masses by the ultrasound machine equipped with a 7-12 MHz probe, using the automatic and semiautomatic 14- and 16-gauge needles by the same radiologist. Five to ten core needle biopsies were collected and they were kept in formalin to be sent to a pathology center in Babol. Indication for surgery was based on the CNB reports and the surgeon's decision. After surgical resection of the masses, they were sent for pathology. The pathological procedures were performed by a single pathologist with more than 30 years of experience.

**Statistical analysis: **After collecting the data, they were analyzed using SPSS software. The descriptive statistics were used for the data analysis. For BI-RADS 3, when a breast mass was established as benign in both imaging and pathology, it was considered as True Positive (TP), and when was determined malignant, it was considered as True Negative (TN). False Positive (FP) was considered when imaging was suggestive of malignancy but histopathology was not consistent, and False Negative (FN) was considered when imaging shows a benign legion but pathology suggested malignancy. For BI-RADS 4A, 4B, 4C, and 5, when a breast mass was established as malignant in both imaging and pathology, it was considered as True Positive (TP), and when was determined benign, it was considered as True Negative (TN). False Positive (FP) was considered when imaging was suggestive of malignancy but histopathology was not consistent, and False Negative (FN) was considered when imaging did not show malignancy but pathology suggested malignancy. Sensitivity was calculated as the proportion of TP to TP+FN, specificity as the proportion of TN to TN+FP, positive predictive value (PPV) as the proportion of TP to TP+FP, negative predictive value (NPV) as the proportion of TN to TN+FN, and accuracy as the proportion of TP+TN to all patients. We also used a receiver operator characteristics (ROC) analysis to estimate the ability of mammography alone, ultrasound alone, and combined mammography and ultrasound to predict breast malignancy, as estimated by the area under the curve (AUC).

**Ethics approval and consent to participate: **The protocol of the present study was approved by the Ethics Committee of Babol University of Medical Sciences (code: MUBABOL.REC.1395.204). The written informed consent was obtained from all research participants after a full explanation of the study. The information of the subjects was kept confidential**.**

## Results

In total, 210 patients underwent CNB, of whom 207 were single and 3 had two breast masses. Overall, 213 breast masses were finally assessed. The mean age of the patients was 46.91±12.22 years old with a range of 19-92 years. The distribution of the patients' mass by CNB results is exhibited in tables 1 and 2. According to pathology, 107 (50.2 %) masses were benign and 106 (49.8 %) masses were malignant.

**Table 1:** Diagnostic value of mammography alone, sonography alone, and combined mammography and sonography for malignant breast masses

**Imaging**	**Sensitivity (%, 95% CI)**	**Specificity (%, 95% CI)**	**Positive predictive value (%, 95% CI)**	**Negative predictive value (%, 95% CI)**	**Accuracy (%, 95% CI)**
Mammography	72.6 (63.1-80.9)	43.9 (34.3-53.9)	56.2 (51.1-61.2)	61.8 (52.7-70.3)	58.2 (51.3-64.9)
Sonography	68.9 (59.1-77.5)	48.6 (38.8-58.5)	57 (51.5-62.4)	61.2 (52.8-69)	58.7 (51.8-65.4)
Mammography+ Sonography	84.9 (76.7-91.1)	43 (33.5-52.9)	59.6 (55.1-63.9)	74.2 (63.5-82.6)	63.9 (57-70.3)

**Table 2:** Diagnostic value of Breast Imaging-Reporting and Data System (BI-RADS) classification for bening and malignant breast masses

**BI-RADS**	**Sensitivity (%, 95% CI)**	**Specificity (%, 95% CI)**	**Positive predictive value (%, 95% CI)**	**Negative predictive value (%, 95% CI)**	**Accuracy (%, 95% CI)**
3	26.2 (18.2-35.)	100 (96.6-100)	100	57.3 (54.5-60)	62.9 (56.1-69.4)
4A	22.6 (15.1-31.8)	22.9 (21.4-39.5)	24.2 (18.1-31.7)	28.1 (22.3-34.7)	26.3 (20.5-32.7)
4B	2.8 (0.6-8.1)	99.1 (94.9-100)	75 (24.1-96.6)	50.7 (49.8-51.7)	51.2 (44.3-58.1)
4C	14.2 (8.1-22.3)	97.2 (92-99.4)	83.3 (59.9-94.4)	53.3 (51.2-55.4)	55.9 (48.9-62.7)
5	60.4 (50.4-69.8)	100 (96.6-100)	100	71.8 (66.8-76.3)	80.3 (74.3-85.4)

**Figure 1:**
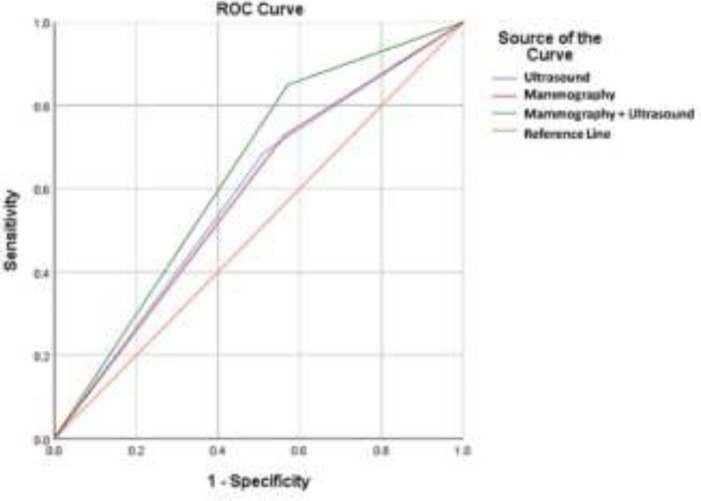
Receiver operating characteristic (ROC) curve of mammography alone, sonography alone, and combined mammography and sonography for predicting breast mass malignancy

**Figure 2:**
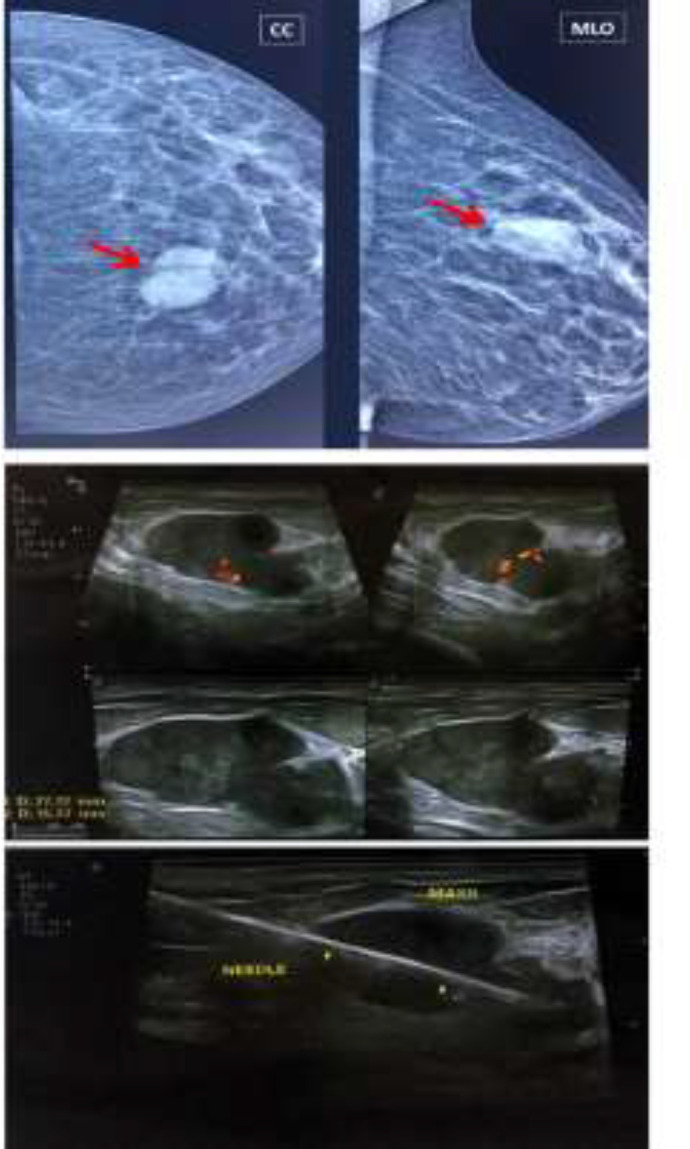
Mammography, ultrasound, and ultrasound-guided core needle biopsy from a left breast mass (BI-RADS 3), which was proved by pathology to be a fibroadenoma

**Figure 3:**
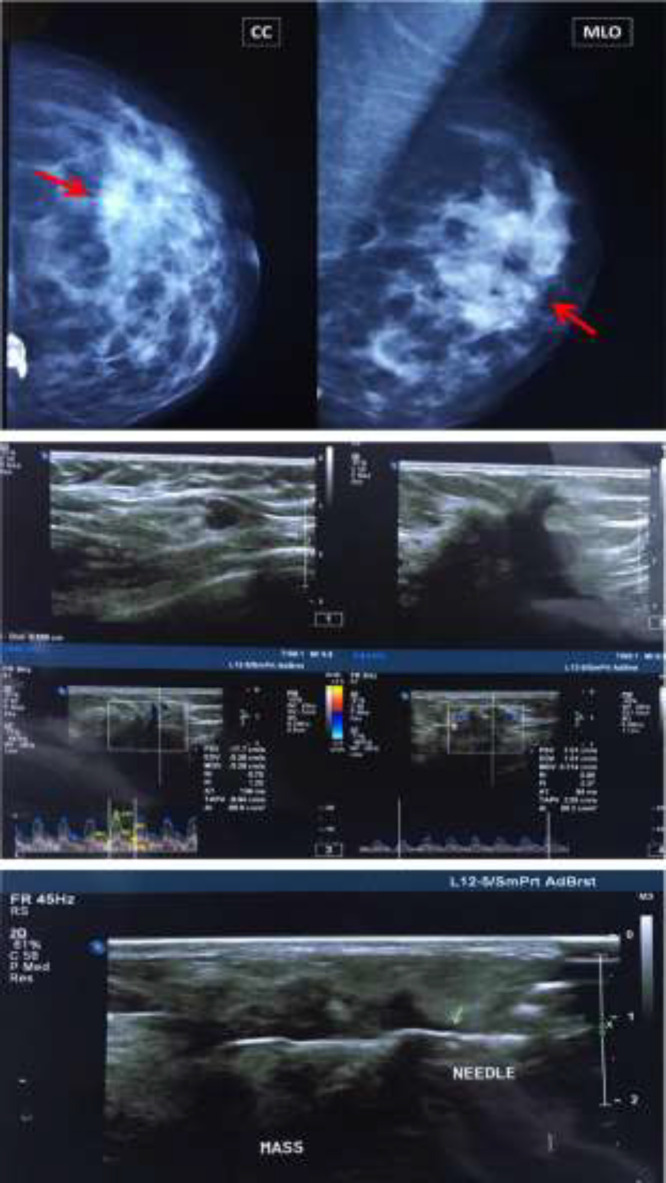
Mammography, ultrasound, and ultrasound-guided core needle biopsy from a left breast mass (BI-RADS 5), which was proved by pathology to be an invasive ductal carcinoma

As shown in table 1, the sensitivity and specificity for mammography alone were 72.6% and 43.9%, respectively. These parameters were 68.9% and 48.6% for ultrasound alone. After a combination of mammography and ultrasound, the sensitivity significantly increased by 12.3% compared with mammography alone and 16% compared with ultrasound alone, reaching 84.9%. On the other hand, the specificity for combined mammography and ultrasound decreased somewhat compared with mammography alone and ultrasound alone. Figure 1 shows the ROC curve for the ability of mammography alone, ultrasound alone, and combined mammography and ultrasound in predicting breast malignancy. The predictive ability of mammography plus ultrasound (AUC=0.639) was higher than that of mammography alone (AUC=0.583) and ultrasound alone (AUC=0.587). Out of all examined masses, 28 masses were classified as BI-RADS 3, 99 as BI-RADS 4A, 4 as BI-RADS 4B, 18 as BI-RADS 4C, and 64 as BI-RADS 5. The computed sensitivity, specificity, PPV, NPV, and accuracy for the individual of BI-RADS classifications are shown in table 2. As indicated, BI-RADS 4A had the highest sensitivity (70.1%) among BI-RADS categories. The highest specificity pertained to BI-RADS 3 and 5 (100%) among BI-RADS categories. Also, the highest accuracy was related to BI-RADS 5 (80.3%). Figures 2 and 3 show mammography, ultrasound, and ultrasound-guided CNB from a benign (BI-RADS 3) and a malignant (BI-RADS 5) breast mass, respectively.

## Discussion

In this study, we evaluated the accuracy of ultrasound and mammography and different BI-RADS classifications in distinguishing breast masses. As found, ultrasound and mammography had a near sensitivity in the diagnosis of breast cancers, but sonography had a higher specificity than mammography. It was also observed that when mammography and ultrasound were combined, the accuracy was higher compared with mammography or ultrasound alone. It means that the combination of mammography and ultrasound would be more helpful in detecting malignant breast lesions compared with that observed for each method alone. On the other hand, specificity for ultrasound plus mammography was lower than mammography or ultrasound alone.

One of the principal challenges of the researchers is which method can be the best way to screen women for breast cancer. Currently, the guidelines recommend women aged at least 40 years old undergo mammography. However, this method had some limitations. For example, mammography is not beneficial enough for women with dense breasts, as its sensitivity is reduced, while women with dense breast tissue have an increased risk of developing breast cancer (11, 12), although there are automatic or semiautomatic systems to aid reporting (e.g., computer-aided diagnosis systems) that allow superior performance to the human reader even in dense breasts in various mammography techniques (13, 14). In this regard, the studies propose to add ultrasound screening to increase breast cancer detection (15).

Some studies have been performed to investigate the accuracy of combined sonography and mammography versus mammography alone. For example, in the study by Berg et al. (16), they stated that sensitivity for combined mammography and ultrasound was 76%, which was higher than that of mammography (52%) or sonography (45%) alone. The authors also reported that specificity for mammography plus ultrasound was 84 %, which was lower than mammography (91%) or sonography (90%) alone (16). Similarly, Buchberger et al. (17) declared that combined screening with mammography and ultrasound for breast cancer increased the sensitivity from 62% (mammography alone) to 81% in women with dense breasts. In other survey by Lee et al. ,(18) who assessed the performance of screening imaging devices for breast cancer in a cohort study, the authors mentioned that additional ultrasound to mammography increased the sensitivity from 74% to 79%, while specificity decreased from 98% to 95%, although these differences were not significant. As observed in the above studies, the sensitivity of mammography increased by supplemental ultrasound, contrary to specificity which decreased (similar to the present study). One of the probable reasons for decreased specificity is that sonography can diagnose some legions that are not detectable by mammography, especially in women with dense breasts, as stated earlier (11, 12). Altogether, the use of ultrasound as a supplemental method for breast cancer screening is still controversial. One of the important reasons is comparably low PPV and/or high NPV concerning the detection of additional malignancies. For instance, the PPV for ultrasound-prompted biopsies has been reported to be in the range of 3.4%–15.9% (17).

In the present study, we also evaluated the accuracy of BI-RADS classification of the imaging techniques. Over recent years, several studies stated that the BI-RADS system could be useful to discriminate malignant and benign breast masses. However, its accuracy rate is still debatable and more results are needed to clearly determine it. In particular, some researchers have compared the BI-RADS accuracy between mammography and ultrasound, and some studies alluded to the comparable accuracy between these two methods (19, 20). Our results showed high rates of PPV for BI-RADS 3-5 categories, which were acceptable. A study reported that ultrasound BI-RADS scoring system could help distinguish between benign and malignant breast lesions, without unreasonably increasing the number of biopsies (21). Overall, BI-RADS 3 refers to masses with regular margin, asymmetric parenchymal densities, and round micro-calcifications. The malignancy risk of BI-RADS 3 is less than 2%, and therefore, most of the specialists recommend a six-month follow-up diagnostic mammography. Regarding BI-RADS 4, the lesions are not classically malignant, however, they are suspicious enough for biopsy. With respect to BI-RADS 5, the lesions have a high malignancy risk and should undergo biopsy. Spiculated masses and clusters of pleomorphic calcifications are classified in this category (5, 22, 23).

Various factors can affect the diagnostic accuracy of imaging methods. Some are related to the patients, such as age, breast surgery history, lesion characteristics, menstrual/menopausal status, and collaboration between patients and technicians in the imaging process, and some are related to the health system, including hardware (i.e., presence of a new standardized device of imaging, like vacuum-assisted breast biopsy technology (24)) and human resources (i.e., presence of an expert radiologist) (25, 26). These factors can explain the differences in the results between the various studies.

A limitation of the present study was that we did not access the CNB results of some patients, so we excluded them from further investigations. Also, the breast density was not specifically considered in stausy design, hence, consideration of this subject would be valuable in the furtehr studies. The results of the present study showed that combined mammography and ultrasound had a higher rate of accuracy than mammography or ultrasound alone in the diagnosis of breast cancers. Furthermore, the imaging methods BI-RADS classification had an acceptable positive predictive value. Altogether, non-invasive diagnostic techniques can be useful in the diagnosis of breast lesions as cost-benefit, easy, and available methods and make the approach to breast lesions easier.
